# Serum neutralizing capacity and T-cell response against the omicron BA.1 variant in seropositive children and their parents one year after SARS-CoV-2 infection

**DOI:** 10.3389/fped.2023.1020865

**Published:** 2023-03-27

**Authors:** Alina Seidel, Eva-Maria Jacobsen, Dorit Fabricius, Magdalena Class, Maria Zernickel, Carmen Blum, Carina Conzelmann, Tatjana Weil, Rüdiger Groß, Sebastian F. N. Bode, Hanna Renk, Roland Elling, Maximillian Stich, Frank Kirchhoff, Klaus-Michael Debatin, Jan Münch, Aleš Janda

**Affiliations:** ^1^ Ulm University Medical Center, Institute of Molecular Virology, Ulm, Germany; ^2^ Department of Pediatrics and Adolescent Medicine, Ulm University Medical Center, Ulm University, Ulm, Germany; ^3^ University Children's Hospital Tuebingen, Tuebingen, Germany; ^4^ Center for Chronic Immunodeficiency (CCI), Medical Center—University of Freiburg, Faculty of Medicine, Institute for Immunodeficiency, University of Freiburg, Freiburg, Germany; ^5^ Center for Pediatrics and Adolescent Medicine, Medical Center, Faculty for Medicine, University of Freiburg, Freiburg, Germany; ^6^ Department of Pediatrics I, University Children's Hospital Heidelberg, Heidelberg, Germany

**Keywords:** SARS-CoV-2, variant of concern, omicron variant, neutralizing antibody, T cells, children

## Abstract

**Introduction:**

Durability of immune protection against reinfection with SARS-CoV-2 remains enigmatic, especially in the pediatric population and in the context of immune-evading variants of concern. Obviously, this knowledge is required for measures to contain the spread of infection and in selecting rational preventive measures.

**Methods:**

Here, we investigated the serum neutralization capacity of 36 seropositive adults and 34 children approximately one year after infection with the ancestral Wuhan strain of SARS-CoV-2 by using a pseudovirus neutralization assay.

**Results:**

We found that 88.9% of seropositive adult (32/36) and 94.1% of seropositive children (32/34) convalescents retained the neutralizing activity against the SARS-CoV-2 Wuhan strain (WT). Although, the neutralization effect against Omicron BA.1 (B.1.1.529.1) was significantly lower, 70.6% (24/34) of children and 41.7% (15/36) of adults possessed BA.1 cross-neutralizing antibodies. The spike 1 (S1)-specific T cell recall capacity using an activation-induced marker assay was analyzed in 18 adults and 16 children. All participants had detectable S1-specific CD4 T cells against WT, and 72.2% (13/18) adults and 81,3% (13/16) children had detectable S1 WT-specific CD8 T cells. CD4 cross-reactivity against BA.1 was demonstrated in all investigated adults (18/18), and 66.7% (12/18) adult participants had also detectable specific CD8 BA.1 T cells while we detected BA.1 S1 reactive CD4 and CD8 T cells in 81.3% (13/16) children.

**Discussion:**

Together, our findings demonstrate that infection with the ancestral strain of SARS-CoV-2 in children as well as in adults induces robust serological as well as T cell memory responses that persist over at least 12 months. This suggests persistent immunological memory and partial cross-reactivity against Omicron BA.1.

## Introduction

Individual immunity is the most crucial factor in containing the ongoing COVID-19 pandemic. Immunity against SARS-CoV-2 can be acquired through infection or by vaccination. Long-lasting protection, however, is challenged by waning immunity over time and the emergence of immune evasive SARS-CoV-2 variants (variants of concern, VOCs) (1). As children and adolescents mainly exhibit mild or even asymptomatic infection, it is of interest to examine the durability of immunity against SARS-CoV-2 in this population. We have recently shown that despite asymptomatic or mild infection, children can mount a potent and durable antibody and cellular response (2, 3).

We now asked whether neutralizing antibodies as well as specific T cells can still be detected one year after infection with ancestral SARS-CoV-2, and what proportion of the seropositive patients show reactivity against the Omicron BA.1 VOC.

## Results

**Figure 1:**
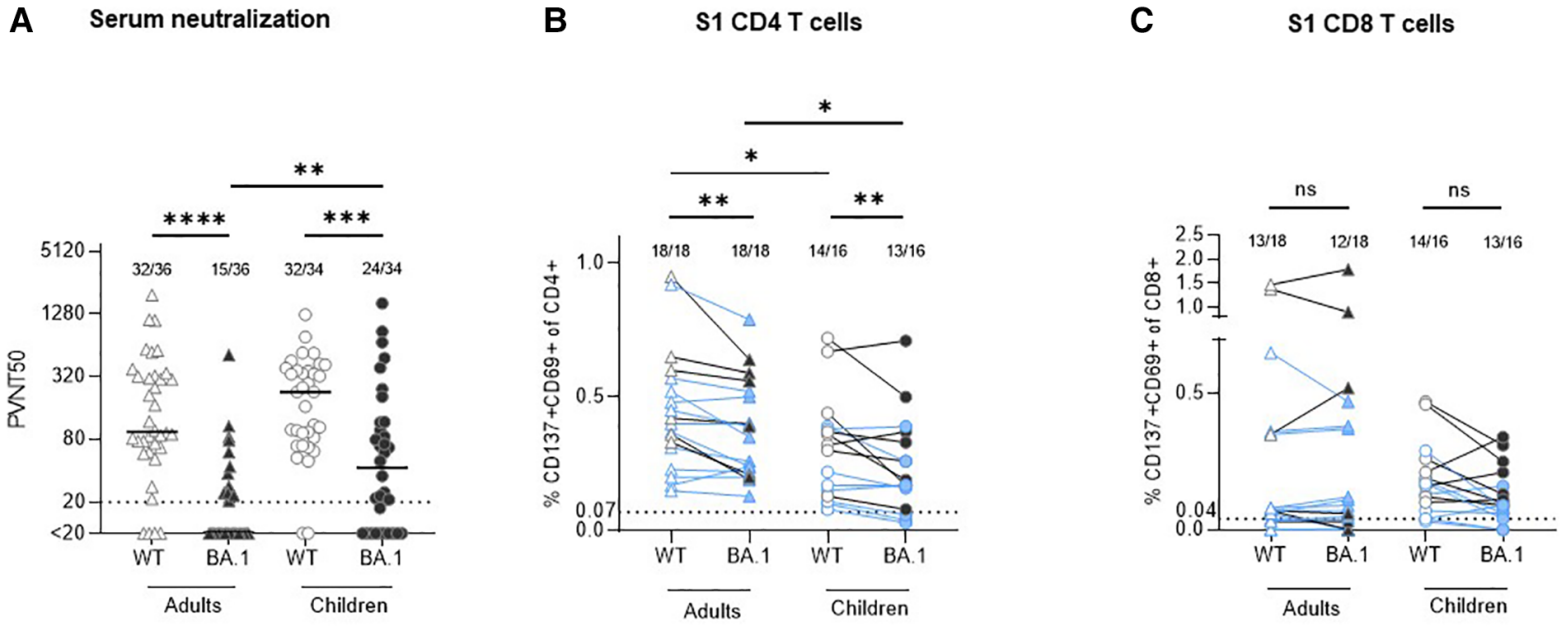
Comparison of serum neutralization capacity and S1-specific T cells against Wuhan strain (WT) and omikron BA.1 (B.1.1.529.1) in adults and children circa 12 months after infection with ancestral SARS-CoV-2 strain. (**A**) Serum neutralization capacity in convalescent adults (triangles) and children (circles) was measured with a pseudovirus neutralization assay against WT (empty symbols) and BA.1 (B.1.1.529.1; filled symbols). The results are presented as serum dilution resulting in 50% pseudovirus neutralization (PVNT50) on cells, calculated by nonlinear regression [(inhibitor) vs. normalized response—variable slope]. The upper and lower cut-off values of this assay were set at PVNT50 > 81.920 and <20 (dotted line), respectively. Median values are plotted with a line. (**B,C**) In all available stored peripheral blood mononuclear cells (PBMC) samples S1-specific CD4 and CD8 T cells were measured with activation-induced marker (AIM) assay using two sets of stimulating peptides (see methods). The same symbols as in the serum neutralization assay are applied; the results from participants with missing neutralization against BA.1 are marked with blue color. The results are presented as percentage of activated T cells (expressing CD137 and CD69) out of the particular T cell subset. The cut-off for positivity (dotted line) was set at 0.07% and 0.04% for CD4 and CD8 T cells, respectively; background values after stimulation with DMSO are subtracted. Numbers of positive samples out of all measured samples are displayed above the particular analysis. The Wilcoxon matched-pairs signed rank test was used for paired samples; Mann-Whitney test was used for the non-paired ones. Statistical significance was defined as ***p ≤ 0.001; only statistically significant differences are marked.

Analysis of a subset of the previously studied serum samples (for patients' characteristics see Table 1) from a household study of adults and children infected with the ancestral SARS-CoV-2 strain (2–4) revealed that about 340 days after infection 88.9% (32/36) of seropositive adult and 94.1% (32/34) of seropositive children convalescents retained neutralizing activity against WT. Children had higher serum neutralization capacity compared with adults (median PVNT50 94,4 [IQR 61.7–324.8] vs. 227.0 [IQR 79.4–372.7], *p* = 0.2622; Figure 1A). The neutralization capacity against BA.1 was significantly lower compared to WT both in adults [median PVNT50 < 20 (IQR < 20–25.10); *p* < 0.0001] and in children [median PVNT50 42.5 (IQR < 20–116.6); *p* = 0.0005], with a significantly higher number of children with BA.1 neutralizing antibodies (70.6%, 24/34) in comparison to adults (41.7%, 15/36; *p* = 0.0037).

**Table 1:** Demographics and key information on the study participants. Samples were taken 114 days (IQR 109–120; T1) and 340 days (IQR 322–356 days; T2) post COVID-19 symptom onset. Only participants who were seropositive at T1 and T2 and showed neutralization capacity against ancestral Wuhan strain at T1 were included; this study concentrates on the analysis of the results at the later (T2) timepoint. Individuals vaccinated against SARS-CoV-2 were excluded. See methods for definition of how samples were defined as being seropositive, asymptomatic or symptomatic. Abbreviations: IQR, interquartile range.

	Adults	Children
Number of participants by age group (*n*)	36	34
Median age (years; IQR)	46.1 (41.8–51.1)	10.6 (8.1–13.3)
Number of females (%)	20 (55.6)	17 (50.0)
Number of asymptomatic (%)	3 (8.3)	12 (35.3)
**Symptoms at disease onset**		
– fever (%)	22 (61.1)	15 (44.1)
– cough (%)	18 (50.0)	10 (29.4)
– dysgeusia (%)	20 (55.5)	4 (11.8)
– diarrhea (%)	7 (19.4)	2 (5.9)
Participants with chronic disease: hypertension, diabetes mellitus, dyslipidemia (%)	7 (19.4)	0

All available cryopreserved peripheral blood mononuclear cells (PBMC) of the study participants were stimulated *in vitro* with a S1-spanning peptide mix of the WT and BA.1 strains, and the percentage of activated T cells (CD137 + CD69+) within the particular population was measured. The results confirm our previous finding (3) that robust CD4 (median 0.41% in adults vs. 0.31% in children, *p* = 0.0452; Figure 1B) and CD8 (median 0.07% in adults vs. 0.17% in children, *p* = 0.3690; Figure 1C) recall T cell responses against the Wuhan strain are present one year after SARS-CoV-2 infection. Of note, in all adult participants who were negative for BA.1 neutralizing antibodies, cross-reactive CD4 T cells against BA.1 S1 were present (12/12), in comparison to only 5 out of 8 (62.5%) children. BA.1 S1-specific CD8 T cells were found in 12 out of 18 (66.7%) adults and in 13 out of 16 (81.3%) children, while also some participants with detectable BA.1 neutralizing antibodies did not have CD8 BA.1 S1 specific T cells (Table 2). In all cases with undetectable CD4 and CD8 T cells against BA.1 both in children as well as in adults, the number of specific T cells reactive to the WT strain was similarly low or undetectable. The number of S1-specific CD4 T cells reactive against WT was significantly higher compared to reactivity against BA.1 both in adults and children with median 0.41% (WT) vs. 0.31% (BA.1; *p* = 0.0025) in adults and median 0.37% (WT) vs. 0.18% (BA.1; *p* = 0.0070) in children. Adults had more S1-specific CD4 T cells against both virus strains compared to children (WT: median 0.41% vs. 0.37%, *p* = 0.0452; BA.1 median 0.31% vs. 0.18%, *p* = 0.0188). None of the differences between the proportion of S1-specific CD8 T cells reactive against WT and BA.1 between children and adults was statistically significant. Thus, although the serological cross-reactivity against BA.1 after infection with the ancestral strain was higher in seropositive children in comparison with adults, the CD4 T cell response against BA.1 seems to be maintained better in adults. The difference in T cell reactivity against BA.1 between the two age groups corresponds well with the degree of reactivity to the ancestral viral strain.

**Table 2:** Presentation of the numeric data of the participants in whom the serum neutralization and S1-specific T cells were measured (18 adults, 16 children) together with age and information on presence of clinical symptoms (see Table 1 for further patients’ characteristics). The positive values (above cut-off) are shown with green, the negative ones with red background. All values correspond with the data in graphs in Figure 1.

(A)																	
Virus strain		**Children**															
	Symptoms	yes	no	yes	yes	yes	no	yes	yes	no	yes	no	yes	no	yes	yes	no
	Age (years)	6	7	7	8	9	9	10	11	11	13	13	13	14	14	14	15
WT	Serum neutralization (PVNT50)	<20	539	422	69	324	343	92	165	104	369	49	336	455	227	69	67
	S1+ (% CD137 + CD69+ of CD4+) T cells	0,17	0,13	0,32	0,15	0,10	0,72	0,11	0,36	0,08	0,67	0,39	0,37	0,30	0,44	0,38	0,22
	S1+ (% CD137 + CD69+ of CD8+) T cells	0,04	0,10	0,16	0,13	0,04	0,46	0,07	0,19	0,03	0,12	0,18	0,21	0,26	0,47	0,17	0,29
BA.1	Serum neutralization (PVNT50)	<20	117	867	<20	<20	69	<20	72	<20	1617	<20	116	683	67	<20	<20
	S1+ (% CD137 + CD69+ of CD4+) T cells	0,17	0,08	0,37	0,17	0,04	0,50	0,06	0,19	0,03	0,71	0,26	0,33	0,26	0,17	0,39	0,16
	S1+ (% CD137 + CD69+ of CD8+) T cells	0,09	0,11	0,21	0,16	0,00	0,25	0,06	0,10	0,00	0,09	0,06	0,34	0,13	0,31	0,04	0,08

(B)																			
Virus strain		**Adults**																	
	Symptoms	no	yes	yes	yes	yes	yes	yes	yes	yes	yes	yes	yes	yes	yes	yes	yes	yes	yes
	Age (years)	22	35	39	40	40	41	43	43	43	45	46	46	47	60	66	40	48	58
WT	Serum neutralization (PVNT50)	322	60	1938	<20	346	86	77	97	587	<20	212	1112	28	73	570	326	67	87
	S1+ (% CD137 + CD69+ of CD4+) T cells	0,48	0,15	0,45	0,20	0,92	0,23	0,31	0,17	0,36	0,57	0,42	0,60	0,37	0,40	0,65	0,95	0,52	0,33
	S1+ (% CD137 + CD69+ of CD8+) T cells	0,04	0,03	0,36	0,06	0,08	0,00	0,07	0,08	0,03	0,65	1,38	0,07	0,06	0,00	0,07	1,47	0,35	0,35
BA.1	Serum neutralization (PVNT50)	<20	<20	<20	<20	<20	<20	<20	<20	28	<20	516	60	<20	<20	107	25	<20	23
	S1+ (% CD137 + CD69+ of CD4+) T cells	0,50	0,13	0,39	0,20	0,79	0,22	0,26	0,24	0,19	0,52	0,40	0,56	0,24	0,40	0,59	0,64	0,35	0,21
	S1+ (% CD137 + CD69+ of CD8+) T cells	0,04	0,05	0,38	0,11	0,12	0,00	0,07	0,09	0,03	0,47	0,90	0,06	0,00	0,00	0,00	1,79	0,37	0,52

## Discussion

In our previous work we showed that children mount a robust SARS-CoV-2 antibody response with high serum neutralization capacity against the ancestral strain, but a lower detectable T cell response in comparison with adults (2, 3). In this study, we demonstrated that sera of about two thirds of the seropositive children and about half of the seropositive adults pose neutralization capacity against the BA.1 variant. This finding is reassuring, however, in contradiction to some previously published data. Chen et al. (5) reported on only 26.7% convalescent children with neutralization capacity against BA.1 about 1,5 months post infection; Sieber et al. (6) stated that children practically lack the neutralizing antibodies against this variant 12 months after SARS-CoV-2 infection. Tang et al. (7) found neutralization capacity against BA.1 in 16% of convalescent children tested 1–3 months after COVID-19. Conversely, analysis of pre-school children with median age of 2 years more than 1 year after SARS-CoV-2 infection proved presence of neutralizing antibodies against BA.1 in 63% of children, but only in 27% of adults (8). Those results are congruent with our findings. The variance between the studies might be possibly caused by different factors. The probably greatest impact had our inclusion criteria that only participants who remained seropositive within the study period were included (see Methods) (2–4). We wanted to know which proportion of the seropositive participants pose also serum cross-neutralization against BA.1 and we deliberately did not test all participants. The differences might be affected by different neutralization assays [live virus microneutralization (5, 6), pseudovirus neutralization assay (7) and plaque reduction (8)] as well (9).

Additionally, we showed that cross-reactivity of S1-WT-specific CD4 and CD8 T cells against the BA.1 variant is present also in convalescent participants with undetectable serum neutralization capacity against this virus variant.

It is highly probable that the cross-reactivity against the further immune escape variants of SARS-CoV-2 (including the currently most widespread BA.5 strain) decreases in children similarly to adults (10).

In conclusion, we proved that children previously exposed to the ancestral strain of SARS-CoV-2 mount a comparable immune response to adults, and the ones who pose SARS-CoV-2 antibodies are partially responding on serological as well as cellular level also to the Omicron BA.1 variant.

## Materials and methods

### Sample collection

For this study, a subset of serum samples and peripheral blood mononuclear cells (PBMC) from a previously described non-interventional, prospective observational national multi-center household cohort study in Southwest Germany (initiated from Universities in Freiburg, Heidelberg, Tübingen, and Ulm) were taken (2, 3). Participants were recruited during the first wave of the pandemic before emergence of SARS-CoV-2 VOCs alpha, beta, gamma, delta and omicron (in Germany July 2020) and first tested at 114.0 days (IQR 109–120) post SARS-CoV-2 infection (T1). The second drawing took place at 340 days (IQR 322–356 days) post SARS-CoV-2 infection (T2) from 101 adults and 80 children of whom 49 (48.5%) adults and 36 (45.0%) children were seropositive. Out of those participants 36 (73.5%) adults and 34 (94.4%) children showed serum neutralization capacity against ancestral Wuhan strain at T1 and were included into the analysis of neutralization capacity against ancestral Wuhan strain and BA.1 VOC as well as specific T-cell recall response at T2 (see Table 1 for participants' characterization). The re-infection during the follow-up was excluded based on the data provided by the participants. This information was supported by falling serum level of antibodies against SARS-CoV-2 nucleocapsid antigen (3,1-fold and 5,4-fold median decrease in adults and children, respectively) between T1 and T2. Antibodies against SARS-CoV-2 were detected using the following four assays: (1) EuroImmun-Anti-SARS-CoV-2 ELISA IgG and IgA (S1), (2) Siemens Healthineers SARS-CoV-2 IgG (RBD), (3) Roche Elecsys Ig (Nucleocapsid Pan Ig) as reported previously (2, 3). Seropositivity was defined as any three of the four SARS-CoV-2 assays being positive. This part of the study was conducted by the University Children's Hospital in Ulm, Germany. Ethics approval was obtained from the respective independent ethics committee (Nr. 152/20). Written informed consent was obtained from adult participants and from parents or legal guardians on behalf of their children. Children's preferences on whether or not to provide a blood sample were respected throughout. This study was registered at the German Clinical Trials Register (DRKS), study ID 00021521, conducted according to the Declaration of Helsinki, and designed, analyzed and reported according to the Strengthening the Reporting of Observational Studies in Epidemiology (STROBE) reporting guidelines.

### Cell culture

Vero E6 (African green monkey, female, kidney; CRL-1586, ATCC, RRID:CVCL_0574) cells were grown in Dulbecco's modified Eagle's medium (DMEM, Gibco) supplemented with 2.5% heat-inactivated fetal calf serum (FCS), 100 units/ml penicillin, 100 µg/ml streptomycin, 2 mM L-glutamine, 1 mM sodium pyruvate, and 1× non-essential amino acids. HEK293T (human, female, kidney; ACC-635, DSMZ, RRID: CVCL_0063) cells were grown in DMEM supplemented with 10% FCS, 100 units/ml penicillin, 100 µg/ml streptomycin, and 2 mM L-glutamine. All cells were grown at 37°C in a 5% CO_2_ humidified incubator.

### Preparation of pseudotyped particles

Rhabdoviral pseudoparticles were prepared as previously described (11). A replication-deficient VSV vector in which the genetic information for VSV-G is replaced by genes encoding enhanced green fluorescent protein and firefly luciferase (12) (kindly provided by Gert Zimmer, Institute of Virology and Immunology, Mittelhäusern, Switzerland) was used for pseudotyping. HEK293T cells were transfected with expression plasmids encoding SARS-CoV-2 spike variants B.1 (ancestral SARS-CoV-2 strain, D614G virus isolate from the early phase of the pandemic) (13) or BA.1 (14) (both plasmids kindly provided by Stefan Pöhlmann, Infection Biology Unit, German Primate Center—Leibniz Institute for Primate Research, Göttingen, Germany). 24 h post transfection, cells were inoculated with VSV vector. After 2 h incubation at 37°C, the inoculum was removed, cells were washed with PBS and fresh medium added. After 16–18 h, the supernatant was collected and centrifuged (2,000 × g, 5 min, room temperature) to remove cellular debris. Cell culture medium containing anti-VSV-G antibody (I1-hybridoma cells; ATCC no. CRL-2700) was added to block residual VSV-G-containing particles. Pseudovirus stocks were then aliquoted and stored at −80°C.

### Pseudovirus neutralisation assay

For pseudovirus neutralisation experiments, Vero E6 cells were seeded in 96-well plates one day prior. Heat-inactivated (56°C, 30 min) sera were serially diluted in PBS, mixed with pseudovirus stocks (1:1, v/v) and incubated for 30 min at 37°C before being added to cells. After 16–18 h, firefly luciferase activity was quantified as a readout for transduction efficiency. For this, cells were lysed by incubation with Cell Culture Lysis Reagent (Promega) at room temperature. Lysates were then transferred into white 96-well plates and luciferase activity was measured using a commercially available substrate (Luciferase Assay System, Promega) and a plate luminometer (Orion II Microplate Luminometer, Berthold). For analysis, background signal of untreated cells was subtracted and values normalized to pseudovirus mixed with PBS only. Results are given as serum dilution resulting in 50% pseudovirus neutralization (PVNT50) on cells, calculated by nonlinear regression [(Inhibitor) vs. normalized response—Variable slope] in GraphPad Prism Version 9.1.1. The upper and lower cut-off values of this assay were set at PVNT50 > 81,920 and PVNT50 < 20, respectively.

### Detection of SARS-CoV-2-specific T cells by activation-induced marker (AIM) assay

Ten to thirty milliliters of peripheral blood were collected in heparin tubes and PBMCs were isolated by density gradient separation (Pancoll separating solution, Pan-Biotech) and cryopreserved for batched analysis. Activation-induced marker (AIM) assay was performed as described before (15–17). PBMCs were thawed in complete RPMI cell culture medium (PAN Biotech), 100 U/ml penicillin/streptomycin, 2 mM L-glutamine and 14 mM HEPES (all Gibco, Thermo-Fisher Scientific) containing 10% FCS (PAN Biotech). After washing twice (PBS, Gibco, Thermo Fisher Scientific), the cells were resuspended at a concentration of 1 × 10E7/ml in complete RPMI cell culture medium containing 10% human AB serum (PAN Biotech). Cells were seeded at 10E6/100 µl/well in a 96-well U-bottom plate and stimulated for four days at 37°C/6.5% CO2 with 1 µg/ml SARS-CoV-2 spike protein peptide-pools: 1. PepMixTM SARS-CoV-2 spike glycoprotein pool 1 (JPT Peptide Technologies), containing overlapping peptides of the N-terminal part (residues 1–643) of the SARS-CoV-2 structural spike glycoprotein, in this study named “S1 (Spike 1)”. Additionally to the ancestral S1 peptide mix, cells were stimulated by a S1 mix of the Omicron BA.1 variant [PepMix™ SARS-CoV-2 (S-RBD B.1.1.529/BA.1/Omicron)].

After four days of stimulation, the cells were washed with PBS and stained with Zombie-green Fixable Live/Dead Stain (Biolegend) for 10 min and RT in the dark, followed by staining with anti-human CD137-PE, CD69-PE Cy7, (all Biolegend), CD3 APC, CD8 APC-AlexaFluor 700, CD4 APC-AlexaFluor 750, and CD45 Krome Orange (all Beckman-Coulter) for 20 min and RT in the dark. Stained cells were washed in IF medium (PBS/5% BSA), and data were acquired on a Beckman-Coulter 10 color NAVIOS Flow cytometer. Data analysis was performed using KALUZA Analysis 2.1. (Beckman-Coulter). Background values of CD137 + CD69+ T cells obtained after DMSO incubation were subtracted from values of CD137 + CD69+ T cells after stimulation with peptide-mixes to calculate the proportion of specifically activated T cells. The limit of detection of specifically activated cells was calculated by assessing the mean +/− standard deviation of unexposed controls (*n* = 25) for CD4 (=0,07%) and CD8 (=0,04%) T cells.

### Statistical analysis

Titres below 20 (lower cut-off value) were set to 10 for statistical analysis (but are shown as <20 in figures). Titres of the same donors against different spike variants were compared by Wilcoxon-Signed-Rank test. Titres of different donors (adults vs. children) were compared by Mann-Whitney-U test. Non-parametric Spearman rank correlation was used to test for associations between titres of single donors against D614G spike and Delta spike. A two-sided alpha error of 5% was applied to analyses. All analyses were done by GraphPad Prism version 9.1.1 for Windows, GraphPad Software, San Diego, California, USA, and R (version 4.0.1).

## Data Availability

The raw data supporting the conclusions of this article will be made available by the authors, without undue reservation.
